# Confocal Analysis of Nuclear Lamina Behavior during Male Meiosis and Spermatogenesis in *Drosophila melanogaster*

**DOI:** 10.1371/journal.pone.0151231

**Published:** 2016-03-10

**Authors:** Fabiana Fabbretti, Ilaria Iannetti, Loredana Guglielmi, Susanna Perconti, Chiara Evangelistella, Luca Proietti De Santis, Silvia Bongiorni, Giorgio Prantera

**Affiliations:** Department of Ecology and Biology, Università della Tuscia, Viterbo, Italy; Universita degli Studi di Roma La Sapienza, ITALY

## Abstract

Lamin family proteins are structural components of a filamentous framework, the nuclear lamina (NL), underlying the inner membrane of nuclear envelope. The NL not only plays a role in nucleus mechanical support and nuclear shaping, but is also involved in many cellular processes including DNA replication, gene expression and chromatin positioning. Spermatogenesis is a very complex differentiation process in which each stage is characterized by nuclear architecture dramatic changes, from the early mitotic stage to the sperm differentiation final stage. Nevertheless, very few data are present in the literature on the NL behavior during this process. Here we show the first and complete description of NL behavior during meiosis and spermatogenesis in *Drosophila melanogaster*. By confocal imaging, we characterized the NL modifications from mitotic stages, through meiotic divisions to sperm differentiation with an anti-laminDm0 antibody against the major component of the *Drosophila* NL. We observed that continuous changes in the NL structure occurred in parallel with chromatin reorganization throughout the whole process and that meiotic divisions occurred in a closed context. Finally, we analyzed NL in *solofuso* meiotic mutant, where chromatin segregation is severely affected, and found the strict correlation between the presence of chromatin and that of NL.

## Introduction

The nuclear envelope (NE) is a cellular ultrastructure that encloses the genetic material in eukaryotic cells. The NE consists of an outer membrane, in continuity with the endoplasmic reticulum, and an inner membrane overlooking the nuclear lumen. In eukaryotes, the inner surface of the NE is lined with a network of filamentous proteins called nuclear lamina (NL) constituted by lamins, which are members of V type intermediate filament family (for review see [1]). The NL provides mechanical support to the NE, and is also involved in important cellular processes such as DNA replication [2] and epigenetic regulation of gene expression [3]. In Drosophila, the NL interacts directly with chromatin at both histone core [4] and DNA specific regions [5]. In higher eukaryotes, the nuclear envelope breakdown at cell division is an important prerequisite for the correct partition of the genetic material into daughter cells.

Two main types of lamins are distinguishable in nature, "A-type" lamins, expressed in a controlled manner during development, and "B-type" lamins, ubiquitously expressed and essential for cellular life. The number and complexity of lamins increase with the evolution of metazoans. *Caenorhabitis elegans* has a single gene for lamins, *lmn-1* [6]. *Drosophila melanogaster* has two genes for lamins, *lmn DM0* and *lmn C*, equivalent to the B-type and A-type genes of vertebrates, respectively. Lamin DM0 is expressed during development, while Lamin C is expressed in late embryo and somatic cells [7–8]. In mammals, three genes were described to encode for lamins, *LMNA*, *LMNB1* and *LMNB2*, which undergo alternative splicing and generate 7 different isoforms [9–12]. In humans, mutations in the *LMNA* gene are associated with several diseases called laminopathies (for a review see [13]). In *Drosophila melanogaster*, lamin Dm0 mutants exhibit severe defects in nuclear envelope assembly, showing the direct role of lamins in the formation of a proper envelope [14]. In mammalian spermatogenesis, the ubiquitous lamin B1, and two germ cell-specific splice variants, lamins C2 and B3 are expressed, whereas lamins A, C and B2 can not be detected [15] [12]. The splice variants C2 and B3 are shorter than the correspondent somatic isoforms, and it was proposed that this feature, together with their low amount in the male meiotic cells, confer the high nuclear flexibility required for the events of spermatogenesis [16–17].

Spermatogenesis is a highly complex differentiation process in which the nuclear architecture dramatically changes from the early spermatogonial stage, passing through meiotic divisions, to the sperm differentiation final stage. Thus, a detailed analysis of NL behavior during spermatogenesis could provide new insights into the regulation of this process. However, a thorough analysis of the NL behavior in male meiosis and spermatogenesis is lacking.

Here, we present the first description of nuclear lamina during *Drosophila melanogaster* meiosis and spermatogenesis. Using confocal microscopy imaging and immunocytology with an antibody against Lamin Dm0, the major component of the Drosophila lamina, we tracked the NL changes throughout spermatogenesis from mitotic phases, through meiotic divisions to sperm differentiation. We found that NL always surrounded the chromatin in all stages of spermatogenesis including the two meiotic divisions, which hence occur in a "closed" context. Moreover, the NL structural changes mirrored the chromatin remodelling that continuously occurs during spermatogenesis, as also shown in a mutant context where chromatin segregation is severely affected. Finally, in the latest stages of sperm differentiation, NL arrangement dramatically changed indicating a possible role of NL in sperm tail patterning.

## Materials and Methods

### Fly strains

*Oregon-R* (Bloomington Drosophila Stock Center, Indiana University) was used as wildtype strian. *βTubulin-GFP/CyO* and *suo*^*1*^*/CyO* fly strains were kindly provided by S. Bonaccorsi and E. Bucciarelli, University of Rome “Sapienza”). Flies were raised on standard *Drosophila* medium at 25°C.

### Cytology

Testes from very young adult males (up to two day-old), were dissected in cold TIB (183mM KCl, 47mM NaCl, 10mM Tris pH 6.8). Testes were transferred in a drop (10 μl) of TIB solution on a microscope slide and covered with a siliconized coverslip. The slide was frozen in liquid nitrogen and the coverslip was removed with a razor blade. Tissues were fixed in cold methanol (-20°C) for 7’ and permealized in PBT (1X PBS, 0.1% Tween20) for 10 minutes. For lamin immunostaning, testes were incubated in a wet chamber, for 1 hour at room temperature, with the monoclonal mouse anti-laminDm0 IgG (Developmental Studies Hybridoma Bank—DSHB- Department of Biological Sciences, University of Iowa, antibody name ADL67.10 [18]) diluted 1:50 in PBT. β-tubulin-GFP chimeric protein was detected by polyclonal rabbit anti-GFP antibody (Torrey Pines Biolabs) diluted 1:200 in PBT. The primary antibodies were detected by 1 hour incubation at room temperature in a wet and dark chamber with either Alexa488-conjugated goat anti-mouse IgG (Molecular Probes) diluted 1:100 in PBT or Alexa594-conjugated goat anti-rabbit IgG (Molecular Probes) diluted 1:200 in PBT. Slides were stained in DAPI (2 mg/ml) in 2XSSC for 10 minutes and mounted in antifade medium (DABCO, Sigma). As a control, testis preparations were incubated with the secondary antibody only (S1 Fig).

### Confocal microscopy

Samples were examined using a Zeiss LSM-710 confocal microscope; images were captured by Zeiss EC Plan-Neofluar 40x/1.30 Oil DIC M27 and Plan-Apochromat 63x/1.40 Oil DIC M27 objective, using ZEN software. Images were processed using ImageJ and Adobe Photoshop.

## Results

### Nuclear lamina localization during premeiotic and meiotic prophase stages

**Fig 1:**
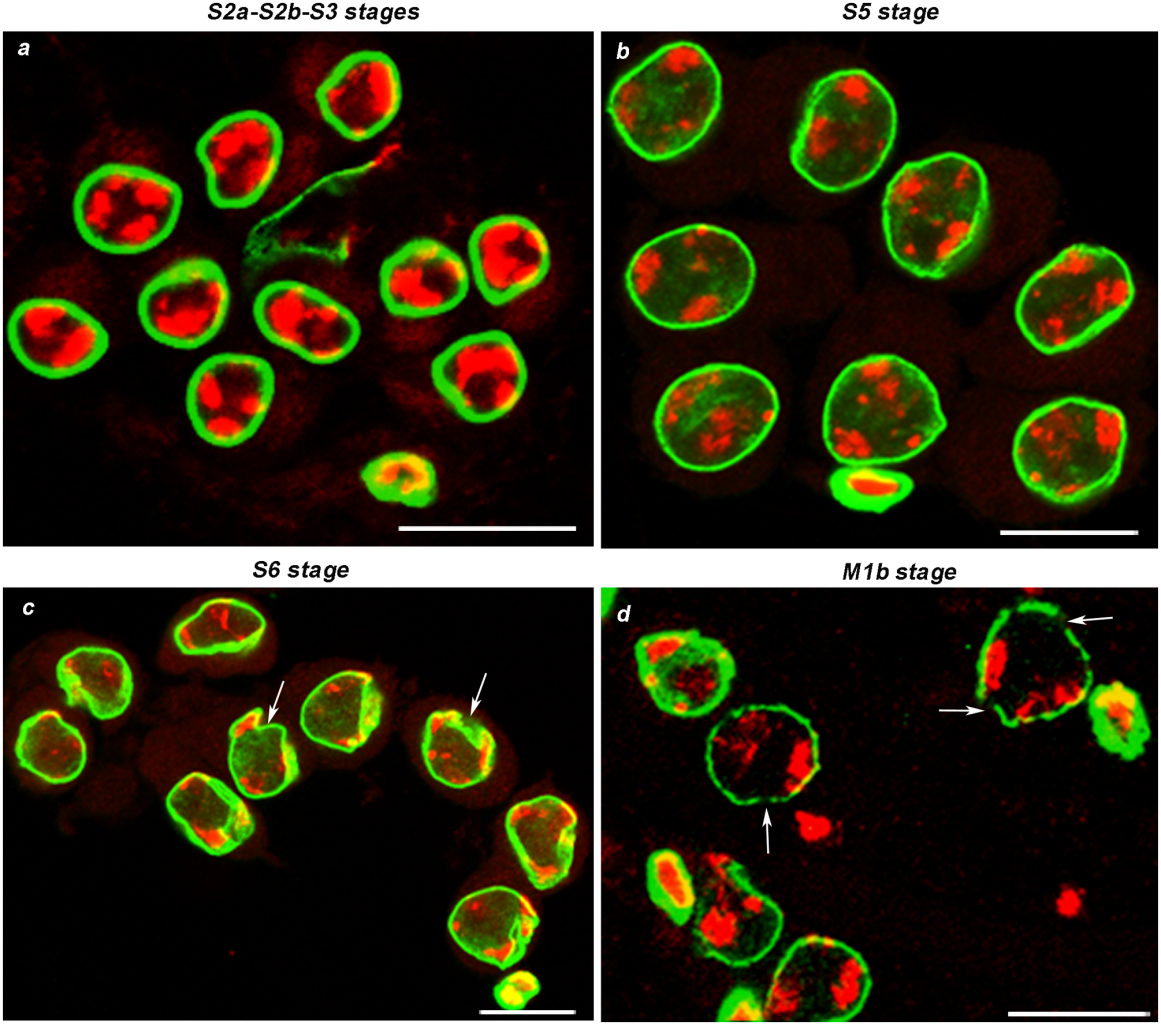
Nuclear lamina behavior in meiotic prophase and prometaphase cells. DNA in red (DAPI staining), nuclear lamina in green (anti Lam-Dm0). In young primary spermatocytes at S2a, S2b and S3 stages (a) and mature primary spermatocytes at S5 stage (b) the NL uniformly depicts the nuclear rim. In primary spermatocytes at S6 stage (c) the NL shows an irregular shape and invaginations (arrows). In prometaphase cells at M1b stage (d), the three chromatin clumps corresponding to the three major bivalents move to the equatorial plate, the nuclear envelope breaks down and the NL signal becomes discontinous (arrows). In a, b, c panels, the heavily lamin-stained cell in the bottom represents a cyst cell. Scale bar 20 μm.

*Drosophila melanogaster* spermatogenesis takes place in testes where a germ line stem cell produces by mitosis a primary spermatogonium (or cystoblast) that undergoes four mitotic divisions giving rise to a cyst of 16 primary spermatocytes [19]. Each primary spermatocyte enters the meiotic programme that leads to the production of 64 haploid spermatids which then develop into mature sperms [19, 20, 21]. We analyzed by confocal microscopy the nuclear lamina (NL) morphological changes throughout spermatogenesis by immunostaining fixed testis preparations with an anti-laminDm0 primary antibody. In *D*. *melanogaster*, *lamDm0* gene is expressed constitutively and at high levels in the testis, where the other lamin gene, *laminC*, is poorly expressed [22]. Thus, we can assume that in the testis the LamDm0 pattern is representative of NL behavior. For the staging of the various spermatogenetic cell types, we mainly refer to the work of Cenci et al. [20]. However, the fixing procedure used in [20] is slightly different from that used in the present study, thus rendering uncertain the identification of the first meiotic division stage. Hence, for a more precise identification of meiosis I stages we immunostained lamina in meiotic cells expressing a GFP-tagged β-tubulin. In premeiotic cells, the NL appeared as a continuous, sharp signal surrounding the nuclei (S2 Fig), which is identical to that observed in early prophase stages (see below). In young primary spermatocytes at S2a, S2b and S3 stages, chromatin was divided in three distinct masses corresponding to the three major bivalents (Fig 1a), and NL appeared as a thick uniform signal encircling the nucleus (Fig 1a). Mature primary spermatocytes at S5 stage, which were recognizable because of their larger nuclear size as compared to young spermatocytes, displayed a NL regular signal comparable to that seen in the preceding stages (Fig 1b). In primary spermatocytes at S6 stage, the lamina signal dramatically changed showing an irregular profile and showing invaginations (Fig 1c, arrows). The analysis of contiguous optical sections revealed that the invaginations at this stage were generally single and very deep (S3 Fig). Prometaphase nuclei at M1b stage were characterized by the equatorial alignment of the three highly condensed major bivalents (Fig 1d). At this stage, the NL signal exhibited a punctuated appearance and showed discontinuities (Fig 1d, arrows).

### Nuclear lamina behavior during meiotic divisions

**Fig 2:**
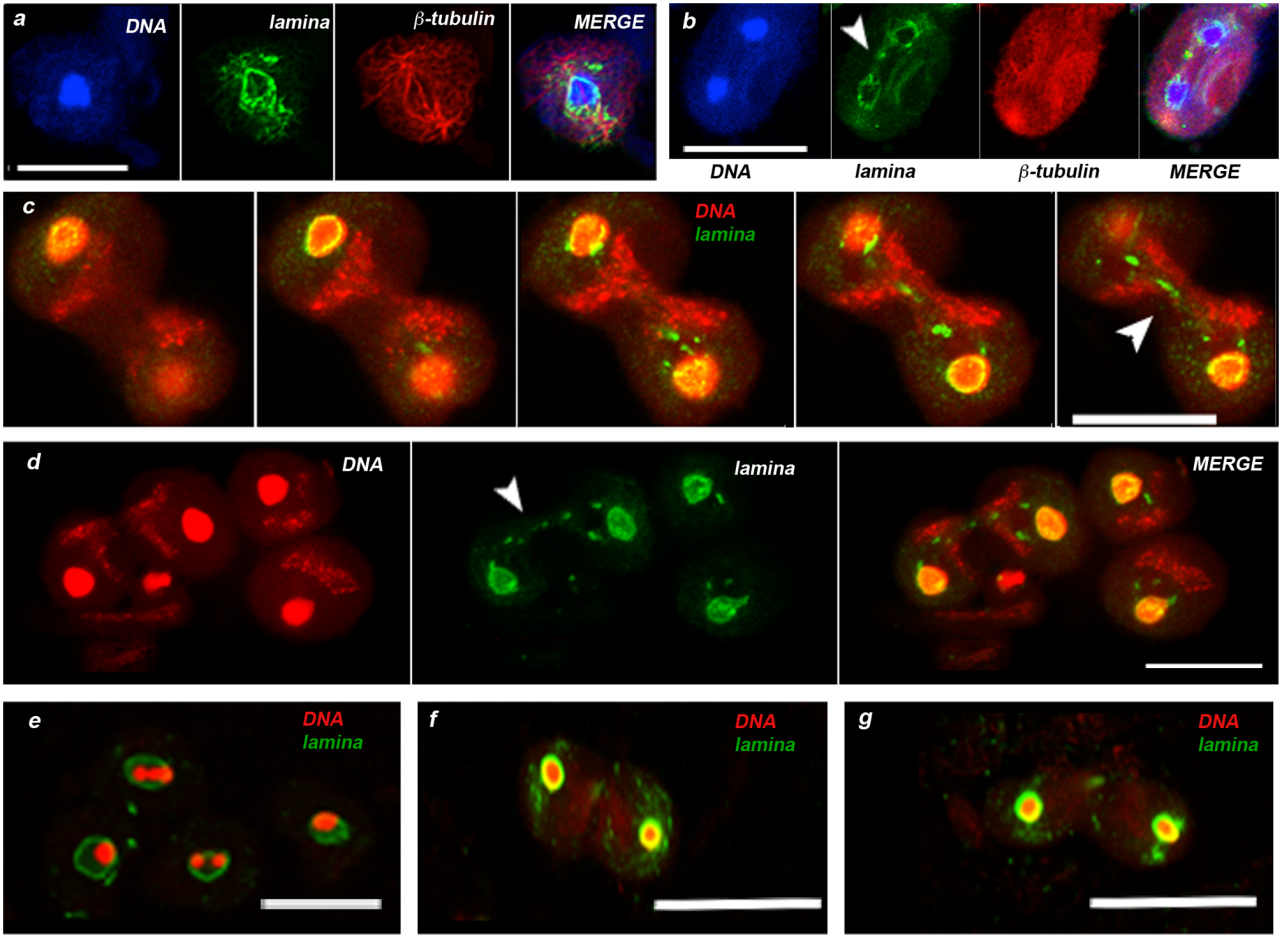
Nuclear lamina pattern during meiotic divisions. In a and b, DNA in blu (DAPI staining), nuclear lamina in green (anti Lam-Dm0) and meiotic spindle in red (anti GFP). (a) Metaphase I, an NL discontinuous signal encircles the nucleus harboring a single condensed chromatin mass. (b) Anaphase I, showing daughter nuclei surrounded by discontinuous NL and a dotted lamin signal associated to the central spindle (arrowhead). In c-g, DNA in red (DAPI staining), nuclear lamina in green (anti Lam-Dm0). (c) Five contiguous optical sections of a mid telophase I, putting in evidence the NL spots associated to the red cloud of mitochondria that demarcates the hourglass-shaped central spindle (arrowhead). Note also that the NL still shows discontinuities. (d) Late telophase I, the nuclear lamina exhibits a thick, irregularly shaped edge. (e) Metaphase II, the chromatin appears either as dots or as unique condensed chromatin mass encircled by NL. (f) Anaphase II and (g) telophase II, the NL shows a well-formed structure encircling nuclear periphery. Scale bar 20 μm.

In *D*. *melanogaster* strain expressing GFP-tagged β-tubulin, NL and GFP were simultaneously detected by the respective antibodies, allowing metaphase I and anaphase I figures to be readily identified. At metaphase I (Fig 2a), the NL showed a rather discontinuous signal encircling the bivalents congregated in metaphase plate (Fig 2a,). At anaphase I (Fig 2b), mid telophase I (Fig 2c) and late telophase I (Fig 2d), the NL signal surrounded the chromatin with an irregularly shaped structure, that in anaphase I figures still showed discontinuities (Fig 2b). In late telophase I, the lamina signal became again continuousand localized also inside the nucleus (Fig 2d). At ana/telophase stages, the NL antibody showed also a spotted signal associated to the central spindle (Fig 2b, 2c and 2d, arrowheads), which was completely absent in the controls lacking the primary antibody (data not shown). During the second meiotic division, the NL apparently did not disaggregate and surrounded the nuclei throughout all the stages from metaphase to telophase (Fig 2d–2f), as it did in the first one. At metaphase II, the chromatin was encircled by a thin irregularly shaped lamin signal, (Fig 2e), whereas in anaphase II (Fig 2f) and telophase II nuclei (Fig 2g) the NL showed a sharp uniform signal at the rim of the nucleus with no spreading over the chromatin (compare merge panel in Fig 2d with those in f and g panel, respectively).

### Nuclear lamina localization pattern in post-meiotic stages of spermatogenesis

**Fig 3:**
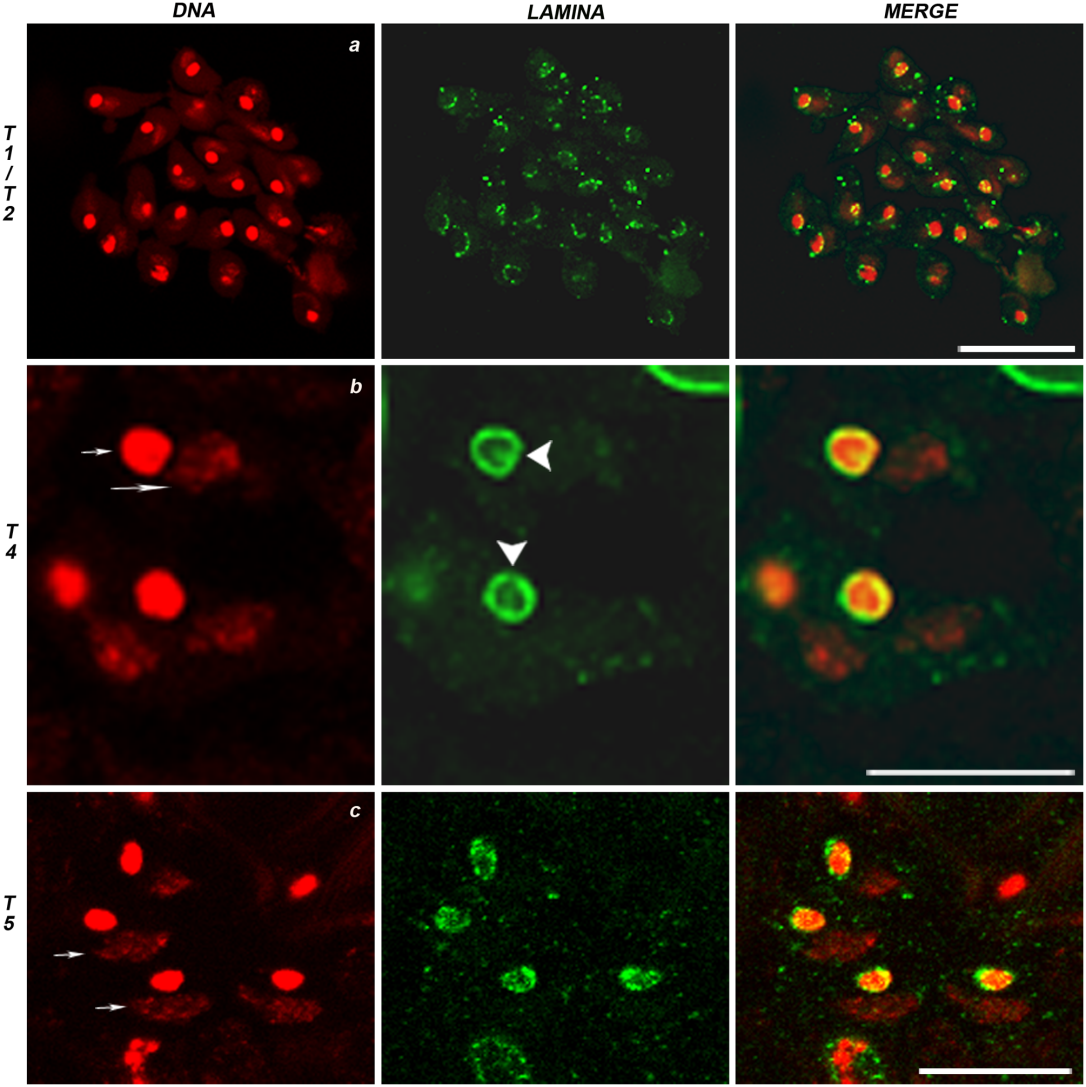
Nuclear lamina distribution during spermatids differentiation. DNA in red (DAPI staining), nuclear lamina in green (anti Lam-Dm0). (a) T1 and T2 stages are characterized by the progressive aggregation of mitochondria into masses of different shapes. NL exhibits a punctuated pattern at the nuclear rim. (b) At onion stage nucleus and Nebenkern (DAPI staining, short and long arrows, respectively), in a 1:1 ratio, have the same round shape and size. NL shows a thick appearance at nuclear periphery, with interruptions (LAMINA staining, arrowheads). (c) T5 spermatid stage is characterized by an oval shaped Nebenkern (DAPI staining, arrows) with NL signal localized also inside the nuclei (c, LAMINA staining and merge). Scale bar 20 μm.

The result of the two meiotic divisions is the formation of a 64 haploid spermatid cyst. Spermatid cells are characterized by the presence of a spherical nucleus associated with a mitochondrial derived organelle, called Nebenkern, composed by several mitochondrial membrane layers (onion stage). At the end of telophase II, mitochondria aggregated forming first an irregular mass of variable shape associated with the nucleus (Fig 3a, DAPI, Stages T1, T2 and T3). At these stages, NL showed an irregular punctuated pattern at the nuclear rim (Fig 3a, LAMINA and merge). At onion stage, with the progress of spermatid differentiation and nuclear condensation (Fig 3b, Stage T4) the NL signal encircling the nucleus became thicker while showing discontinuities (Fig 3b, LAMINA, arrowheads). At T5 stage (Fig 3c), when spermatid chromatin undergoes decondensation and Nebenkern assumes an oval shape (Fig 3c, arrows in DAPI), the NL signal appeared not only at the rim but also inside the nuclei (Fig 3c, LAMINA and merge).

**Fig 4:**
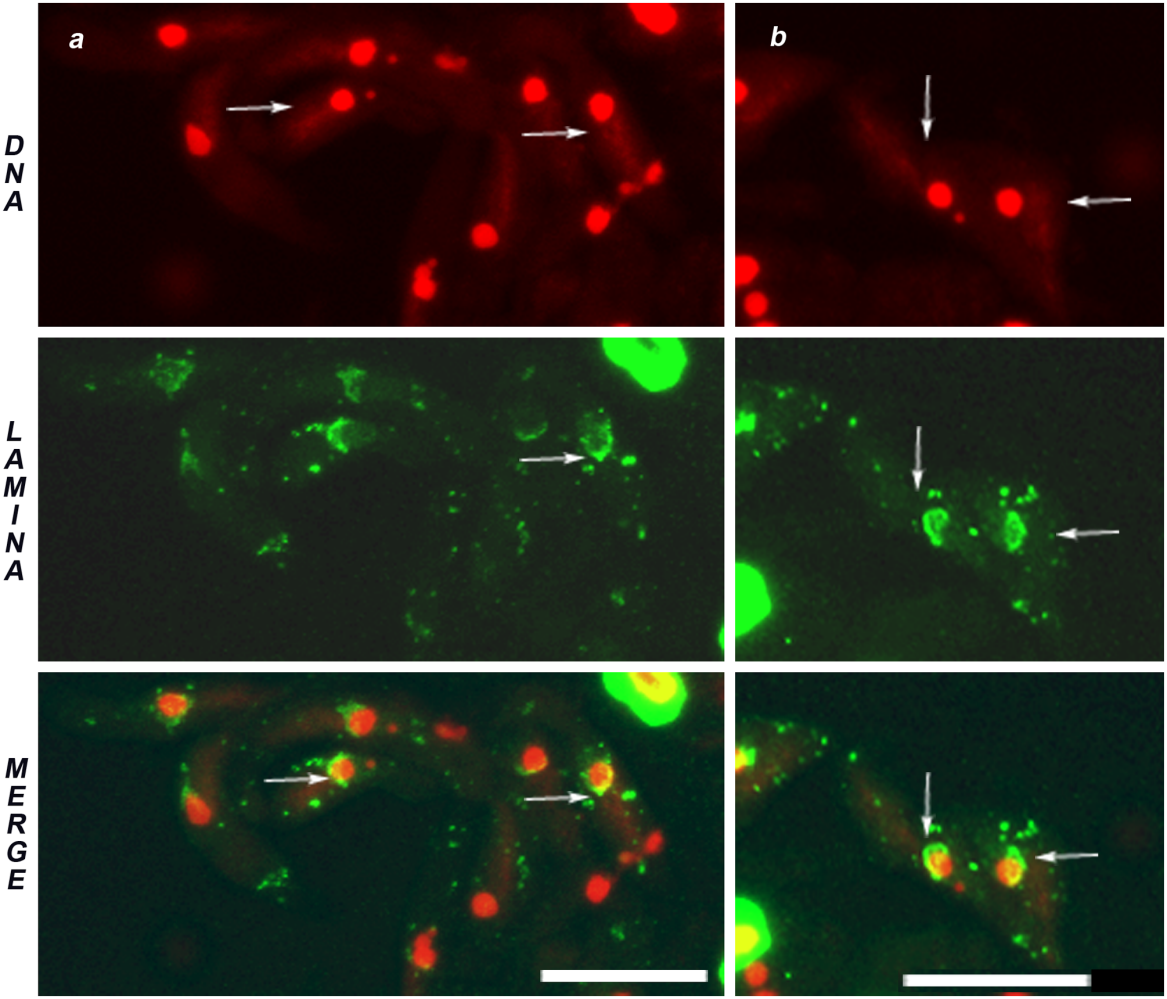
Nuclear lamina distribution during the spermatid elongation. DNA in red (DAPI staining), nuclear lamina in green (anti Lam-Dm0). Note the “half moon” configuration of NL that localizes from the same nuclear side of the elongating Nebenkern (arrows). Scale bar 20 μm.

During spermatid elongation process, chromatin condensed again, and Nebenkern elongated forming the primordium of the future sperm tail (Fig 4). At this stage, the NL signal dramatically changed becoming localized only at the side of the nucleus from which the tail lengthened thus assuming a “half moon” configuration (Fig 4, arrows) The spermatogenesis process culminates in the differentiation and maturation of sperms, (S4 Fig, DAPI staining). The mature sperms heads were completely devoid of NL signal (S4 Fig).

### Nuclear lamina behavior during *solofuso* mutant meiotic and postmeiotic stages

**Fig 5:**
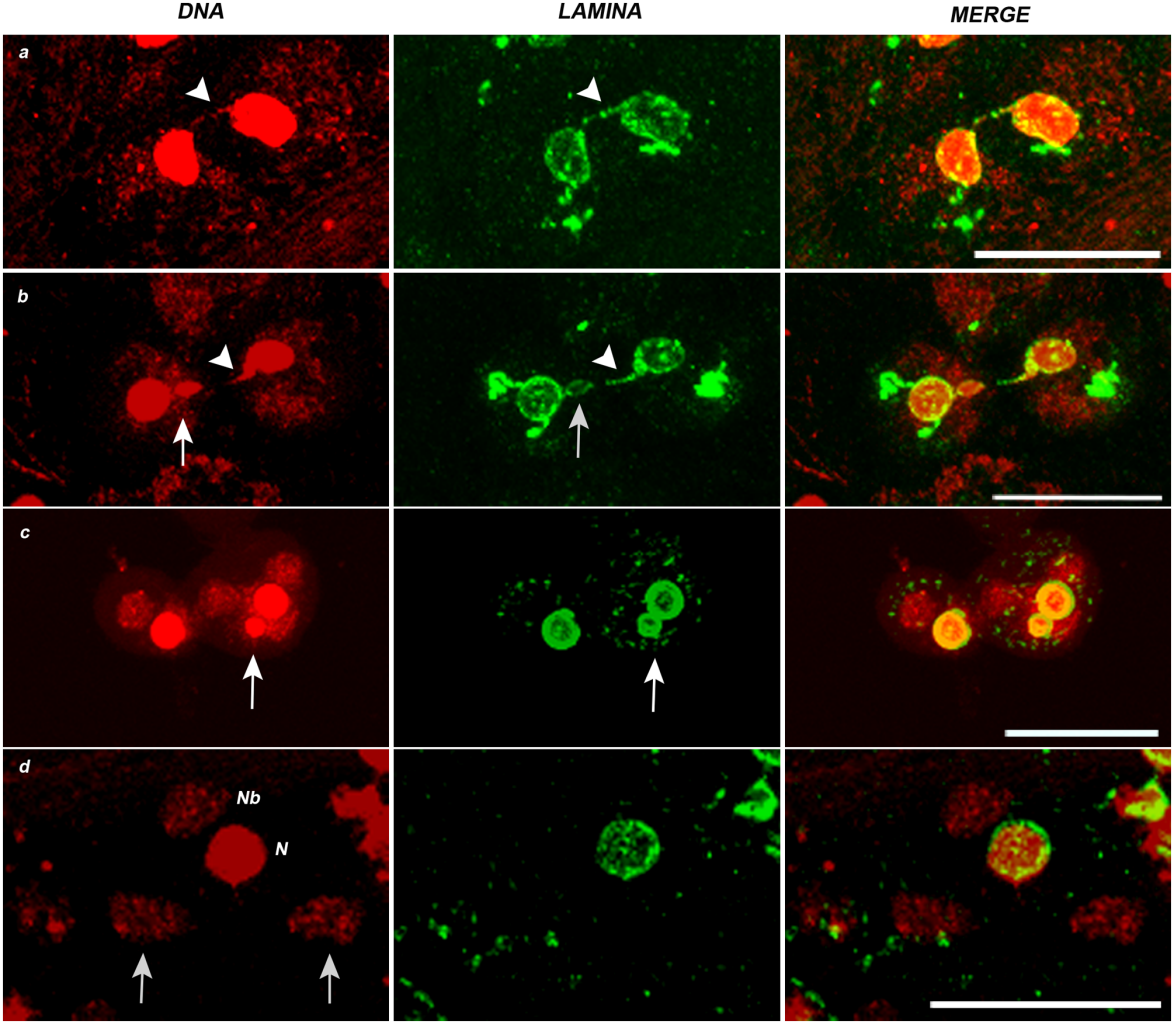
Nuclear lamina behavior during *solofuso* mutant meiotic and postmeiotic stages. DNA in red (DAPI staining), nuclear lamina in green (anti Lam-Dm0). *solofuso* mutant males show chromatin bridges at (a) Anaphase I (arrowheads) and (b) Telophase I (arrowheads). NL shows an irregular signal at nuclear rim and a punctuated pattern over the chromatin bridges (a and b, arrowheads). Note that NL encircles the micronucleus (b, arrows). (c) Spermatids at onion stage evidencing a micronucleus surrounded by NL. (d) Onion stages showing a Nebenkern (Nb) associated to a nucleus (N) and two Nebenkern not associated with nuclei (d, DNA, arrows). The NL signal is detected around the nucleus associated with Nebenkern while no structured NL is present close to the Nebenkern lacking nuclei (d, LAMINA). Scale bar 20 μm.

The above results showed that the NL changes during spermatogenesis accompanied the nuclear chromatin remodeling throughout the process. This correlation suggested us to investigate the organization of NL in Drosophila *solofuso* (*suo*) mutant which results defective in chromosome segregation and generates chromatin free dividing nuclei [23]. *suo* ana-telophases I are characterized by the presence of chromatin bridges ([23]; present paper Fig 5a and 5b, arrows in DAPI). This chromosome missegregation leads to the formation of unbalanced secondary spermatocytes and spermatids ([23]; present paper Fig 5c and 5d, DAPI). In the most extreme cases *suo* onion stage spermatids are characterized by the presence of Nebenkern not associated to any nucleus ([23]; present paper Fig 5d DAPI, arrows) originating from the second meiotic division of secondary spermatocytes devoid of chromosomes [23]. In the mutant meiotic cells, the NL behavior was similar to that of the wild type with two peculiarities. First, the NL signal appeared associated to the ana-telophase chromatin bridges with a punctuated pattern (Fig 5, arrowheads in a and b) and encircled the ensuing micronuclei (Fig 5, arrows in b and c). Second, in mutant onion stages (Fig 5c and 5d), the NL signal regularly encircled the nuclei associated to Nebenkern, but notably no structured lamina signals could be detected in the proximity of Nebenkern not associated to nuclei (Fig 5d, arrows).

## Discussion

Lamin family proteins are nuclear lamina (NL) components that are essential for its crucial role in shaping and supporting the cell architecture. In this context, it results very intriguing to observe how NL changes during spermatogenesis in which the nuclear architecture dramatically changes from the early mitotic spermatogonial stage, passing through meiotic divisions, and ending with the sperm differentiation. However, few data are reported in the literature regarding the NL behavior in male meiosis and spermatogenesis. To fill this gap we undertook a detailed scrutiny of NL changes during spermatogenesis in *Drosophila melanogaster* by confocal imaging of germ cell cytological preparations stained with an anti lamin DmO antibody.

The NL pattern during mitotic spermatogonial stages and the prophase of the first meiotic division closely resembles that of embryonic mitotic cells. Specifically, in *Drosophila* embryonic mitoses, interphase and prophase nuclei showed an intact NL at nuclear periphery [24], and changes in the nuclear envelope structure appeared at late prophase/metaphase transition [25–27]. At this stage, invaginations of NL became detectable in regions close to centrosomes [28]. Similarly, we could show that in meiosis I deep invaginations formed in NL at the transition prophase/metaphase, and gaps in its structure became apparent during chromosome congression to meiotic metaphase plate. This meiotic lamina behavior resembles also to that of mammalian mitotic cells where gaps in the nuclear enevelope structure appeared in connection with chromosome congression [29]. However, we never observed NL disassembly in meiosis I.

Specific to meiosis, moreover, is the lamin association with central spindle at ana-telophase I, which could be functional to ensure an equal partition of NL components between daughter cells allowing the prompt restoration of a complete NL around daughter nuclei that immediately enter the second meiotic division.

The NL behavior during the second meiotic division resembled that of the first one. The NL, in fact, did not disappear and encircled nuclei throughout the whole second meiotic division.

Higher eukaryotes are, in general, characterized by an open mitosis where nuclear pore complexes and lamina disassembled in prophase, concomitantly with nuclear envelope breakdown, and reassembled around telophase nuclei [30]. In *Drosophila* embryo mitotic cells the lamina signal localized exclusively at nuclear periphery up to the metaphase and the lamin delocalization process was completed only when chromosomes moved to anaphase, with the NL reorganization taking place at early mitotic interphase [28]. This pattern was named as semi-open mitosis [28]. In lower eukaryotes, instead, the mitosis is closed, with the chromosome segregation events occurring within an intact nuclear envelope [31]. To our knowledge, such a classification of the NL pattern does not exist for meiosis. Our observations highlight that male meiosis in *Drosophila* occurs in a "closed" context. In fact, NL reorganized throughout the whole meiotic process but never disassembled, thus fully differing from what described for *Drosophila* mitosis by Paddy et al. (1996) [28].

A structured lamina during cell divisions seems to be a mandatory condition to ensure the correct chromosome condensation and behavior in mitosis. In *Caenorhabditis elegans* the downregulation of the lamin gene, *lmn-1*, produces defects in chromatin condensation and homolog segregation [6]. In mammalian mitotic cells, Lamin B associates to chromosomes during metaphase congression [32]. In *Drosophila*, evidences indicate that the NL plays a role in chromatin organization [33]. A number of evidences were accumulated showing that lamin proteins interact with specific DNA sequences, the matrix attachment regions [5], as well as with chromatin components, such as the histone core [4]. Moreover, a proper organization of NL structure is required in flies to achieve a correct localization of the heterochromatin protein HP1 [33], which in culture cells was shown to be involved in the formation of a new nuclear envelope around the daughter nuclei [34]. Recently, a model of nuclear architecture in which lamins position the chromosomes in the nucleus was proposed [35].

This suggested connection between chromatin organization and NL is strongly supported by our observations of the post-meiotic stages of spermatogenesis. Indeed, at the end of meiotic divisions, spermatids enter into a differentiation program characterized by a series of chromatin condensation and decondensation events [20], which are accompanied by modifications of NL pattern. At the early stage of spermatid differentiation, where mitochondria aggregated and chromatin started to condense, the NL lost the compact appearance of the previous stage and exhibited a punctuated pattern. At onion stage, the highly condensed nucleus was encircled by a well-structured, thick, lamina signal that presented small gaps. Finally, a diffused intranuclear distribution of the lamina accompanied the chromatin decondensation that, together with Nebenkern elongation, characterizes the late stages of spermatid differentiation. This apparent ability of the NL to reorganize in parallel with chromatin reorganization emphasizes again the strong dynamics of this structure. The observed pattern is in accordance with functions of nuclear lamins in determining and maintaining the nuclear shape (for a review see [1]). In fact, the chromatin condensation/decondensation events imply nuclear conformational changes that may likely require NL reorganization. In mammals, the differences in the composition of lamins of spermatogenetic cells with respect to its somatic counterpart may reflect the nuclear organization changes during spermatogenesis. Mammalian germ line-specific lamins B3 and C2 are shorter and in minor amounts, with respect to their somatic counterparts, thus allowing the formation of a more flexible NL structure, suited to address the nuclear changes characterizing gametogenesis (For a review see [36]).

The strict correlation between chromatin and NL at meiosis is further corroborated by the NL behavior during meiosis of the male sterile mutant *solofuso* [23]. NL spots decorate the anaphase chromatin bridges that form in the homozygous mutant, then the lagging chromatin become promptly encircled by a consistent, continuous NL rim thus appearing as a micronucleus. Moreover, no NL signal can be detected in chromatin devoid spermatids containing only Nebenkern. These observations suggest that the presence of chromatin is a necessary condition for the organization of a NL rim.

Toward the end of spermatid elongation, the NL positioned asimmetrically at the side of nucleus where the spermatid tail begins to elongate. Similarly, in mouse, lamin B3 polarized at the posterior pole of elongating spermatid nuclei [36]. The strikingly analogous pattern of NL in *Drosophila* and mouse late differentiating spermatids (compare Fig 4, upper row of the present paper and Fig 4D’ of the Schutz’s et al. paper [36]) suggests a functionally relevant role for this conserved, polarized pattern of lamin proteins at the late stage of spermiogenesis. In mammalian post meiotic stages, several nuclear envelope associated proteins polarized to the posterior pole of spermatids and then result undetectable in mature sperms [37], as it does laminDm0 in our study. This behavior was observed for proteins that directely or indirectly interacted with chromatin as LAP2, lamin B1 [37] and lamin B receptor (LBR) [38]. Due to the ability of LAP2, lamin B1 and LBR to bind chromatin, it is plausible to speculate that the redistribution of these nuclear envelope proteins to the posterior pole of spermatids, would contribute to the achievement of non-random chromatin organization in the mature sperm [37]. In this context it is worth to mention that at onion stage the nucleoporin RAE1 redistributes over the nebenkern that localized to the posterior pole of mature spermatids and that rae1 mutations produce male sterility [39].

The general overview emerging from our results is that during spermatogenesis NL continuously reorganizes starting from meiotic prophase until the end of spermatogenesis. These structural changes run in parallel with the rounds of chromatin condensation/decondensation throughout the spermatogenesis process. Our observations are in line with the pioneering immunofluorescence data describing changes of NL distribution throughout the mitotic cell cycle [40–41]. Our results strongly argue in favor of a reciprocal interaction between nuclear lamina and chromatin being the latter necessary for the organization of the former, which in turn accompanies the chromatin remodeling cycles.

## Supporting Information

S1 Fig Testis preparations staining by secondary antibody. In the absence of a previous anti Lam-Dm0 staining, the Alexa488-conjugated goat anti-mouse IgG secondary antibody (right panel) did not produce any specific staining of the primary spermatocytes shown in left panel. For comparison see Fig 1. Note that the secondary antibody image was adjusted to a very high brightness value to obtain a faint staining of cells thus allowing the comparison with the DAPI staining. Scale bar 20 μm.
(TIF)

S2 Fig Nuclear lamina in early stages of spermatogenesis. DNA in red (DAPI staining), nuclear lamina in green (anti Lam-Dm0). A testis apex containing the early stages of spermatogenesis, germ line stem cells, cystoblasts and spermatogonia. The nuclear lamina signal surrounds all the nuclei of the testis apex. Scale bar 20 μm.
(TIF)

S3 Fig Nuclear lamina dynamics in late prophase spermatocytes. DNA in red (DAPI staining), nuclear lamina in green (anti Lam-Dm0). Six contiguous optical sections showing three late primary spermatocytes with NL invaginations (each indicated by different arrows). Confocal analysis shows that all the invaginations are very deep and that two out of three are single. Scale bar 20 μm.
(TIF)

S4 Fig Nuclear lamina in mature sperms. DNA in red (DAPI staining), nuclear lamina in green (anti Lam-Dm0). The nuclear lamina signal is completely absent from the needle-shaped sperm heads and from sperm tails. Scale bar 20 μm.
(TIF)
