# Supplementary figures and images for: Confocal Analysis of Nuclear Lamina Behavior during Male Meiosis and Spermatogenesis in Drosophila melanogaster
Source: PLoS One. 2016 Mar 10;11(3):e0151231. doi: 10.1371/journal.pone.0151231 (PMC4786128; doi:10.1371/journal.pone.0151231)

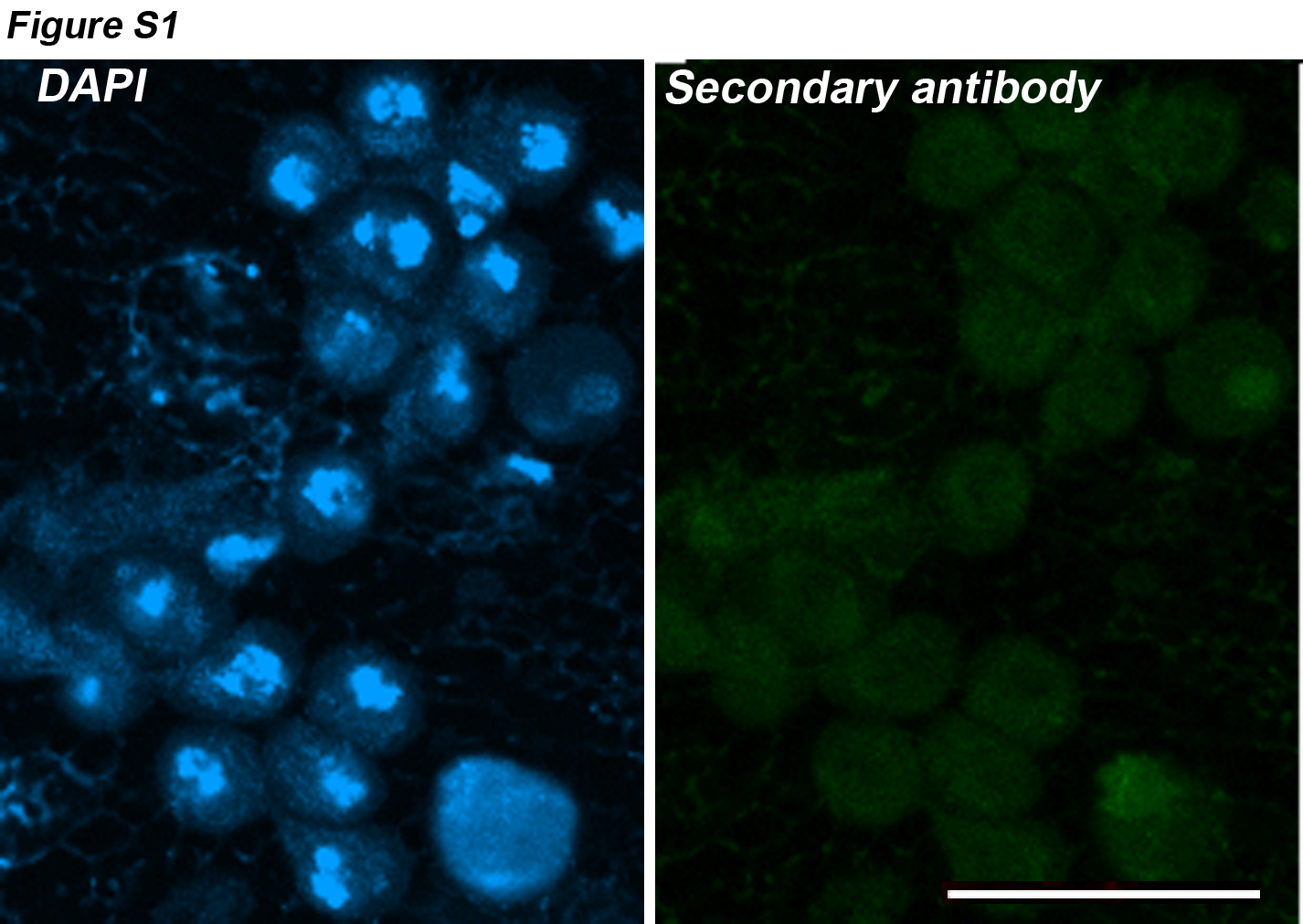

Supplement: S1 Fig — In the absence of a previous anti Lam-Dm0 staining, the Alexa488-conjugated goat anti-mouse IgG secondary antibody (right panel) did not produce any specific staining of the primary spermatocytes shown in left panel. For comparison see Fig 1. Note that the secondary antibody image was adjusted to a very high brightness value to obtain a faint staining of cells thus allowing the comparison with the DAPI staining. Scale bar 20 μm. (TIF) [file pone.0151231.s001.tif]

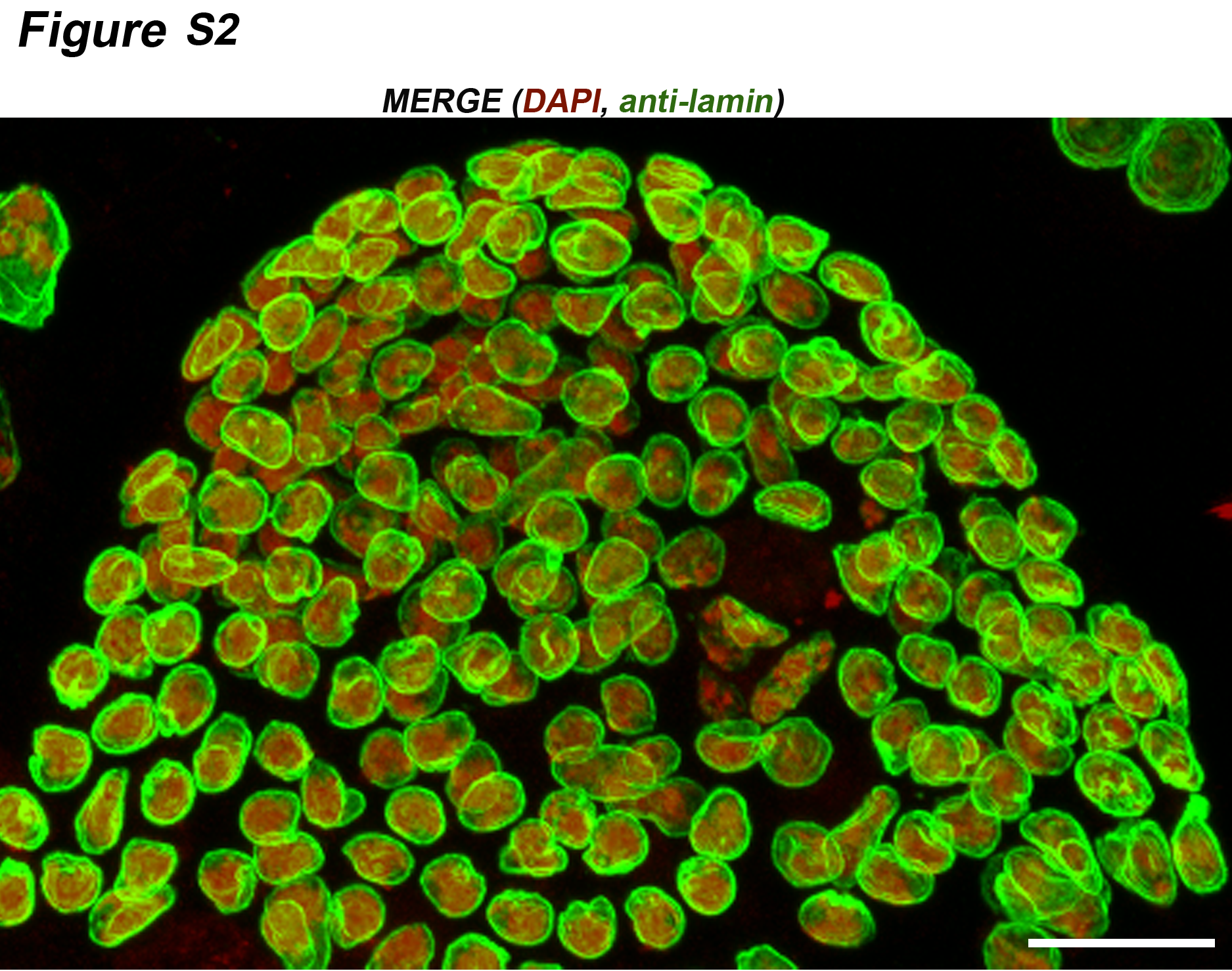

Supplement: S2 Fig — DNA in red (DAPI staining), nuclear lamina in green (anti Lam-Dm0). A testis apex containing the early stages of spermatogenesis, germ line stem cells, cystoblasts and spermatogonia. The nuclear lamina signal surrounds all the nuclei of the testis apex. Scale bar 20 μm. (TIF) [file pone.0151231.s002.tif]

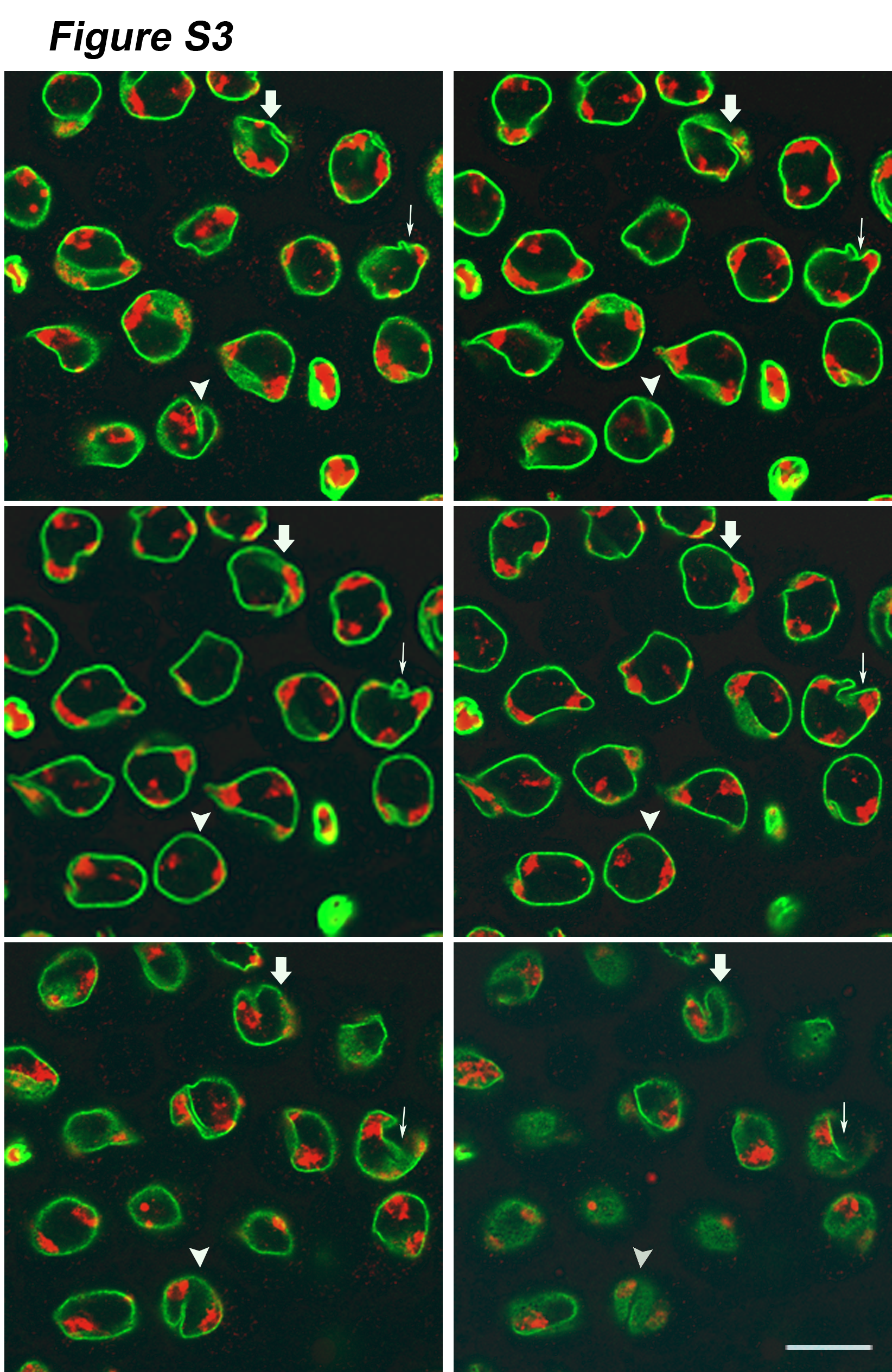

Supplement: S3 Fig — DNA in red (DAPI staining), nuclear lamina in green (anti Lam-Dm0). Six contiguous optical sections showing three late primary spermatocytes with NL invaginations (each indicated by different arrows). Confocal analysis shows that all the invaginations are very deep and that two out of three are single. Scale bar 20 μm. (TIF) [file pone.0151231.s003.tif]

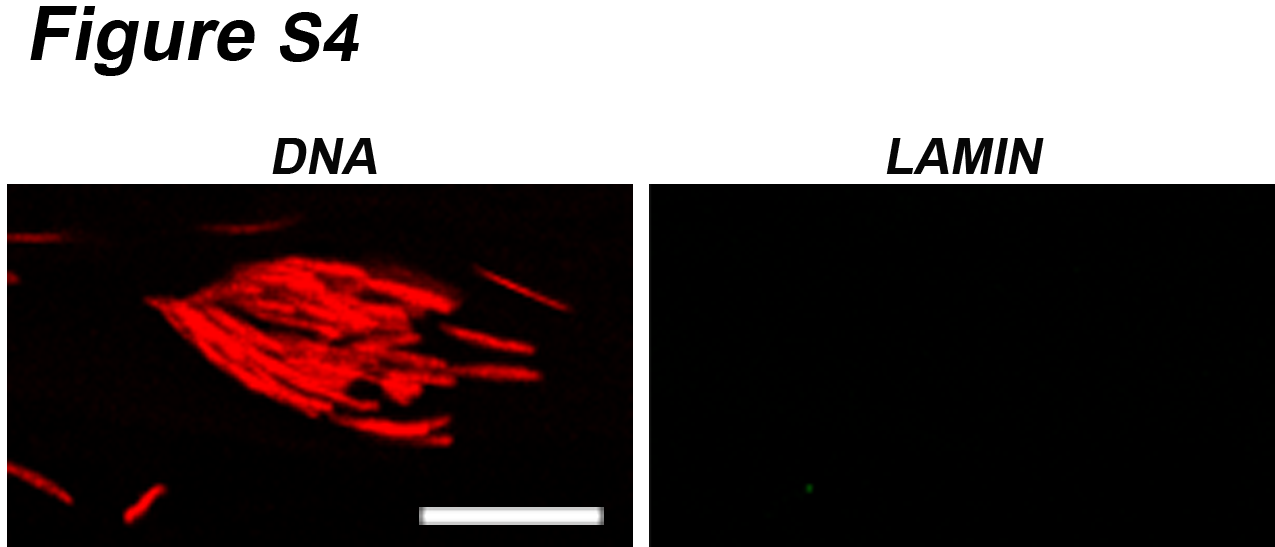

Supplement: S4 Fig — DNA in red (DAPI staining), nuclear lamina in green (anti Lam-Dm0). The nuclear lamina signal is completely absent from the needle-shaped sperm heads and from sperm tails. Scale bar 20 μm. (TIF) [file pone.0151231.s004.tif]
